# Targeted Magnetic Nanotheranostics of Cancer

**DOI:** 10.3390/molecules22060975

**Published:** 2017-06-12

**Authors:** Irina Belyanina, Olga Kolovskaya, Sergey Zamay, Ana Gargaun, Tatiana Zamay, Anna Kichkailo

**Affiliations:** 1 Krasnoyarsk State Medical University named after prof. V.F. Voino-Yaseneckii, 660022 Krasnoyarsk, Russia; irina.belyanina2208@gmail.com (I.B.); olga.kolovskaya@gmail.com (O.K.); tzamay@yandex.ru (T.Z.); 2 Federal Research Center, KSC Siberian Branch of Russian Academy of Science, 660022 Krasnoyarsk, Russia; sergey-zamay@yandex.ru; 3 Independent Researcher Vancouver, Vancouver, BC V6K 1C4, Canada; ana.gargaun@gmail.com

**Keywords:** magnetic nanoparticles, aptamers, drug delivery, magnetodynamic therapy, magnetic hyperthermia, magnetophoresis

## Abstract

Current advances in targeted magnetic nanotheranostics are summarized in this review. Unique structural, optical, electronic and thermal properties of magnetic materials in nanometer scale are attractive in the field of biomedicine. Magnetic nanoparticles functionalized with therapeutic molecules, ligands for targeted delivery, fluorescent and other chemical agents can be used for cancer diagnostic and therapeutic purposes. High selectivity, small size, and low immunogenicity of synthetic nucleic acid aptamers make them attractive delivery agents for therapeutic purposes. Properties, production and functionalization of magnetic nanoparticles and aptamers as ligands for targeted delivery are discussed herein. In recent years, magnetic nanoparticles have been widely used in diagnostic methods, such as scintigraphy, single photon emission computed tomography (SPECT), positron emission tomography (PET), magnetic resonance imaging (MRI), and Raman spectroscopy. Therapeutic purposes of magnetic nanoconstructions are also promising. They are used for effective drug delivery, magnetic mediated hypertermia, and megnetodynamic triggering of apoptosis. Thus, magnetic nanotheranostics opens a new venue for complex differential diagnostics, and therapy of metastatic cancer.

## 1. Introduction

Magnetic nanotheranostics in the last few decades has been an area of priority in biomedicine, specifically for the treatment of various cancers. The greatest interest in oncology is the application of nanostructures with high colloidal and photothermal stability, that exhibit a low percentage of non-specific binding to the biological sample, and have low toxicity for the organism [1]. These include various inorganic gold and magnetic nanoparticles (NPs), lipid NPs, liposomes, quantum dots, dendrimers, polymer nanoparticles such as micelles, and dendrimer structures [2,3,4,5,6,7,8]. Of particular interest are nanoparticles possessing magnetic properties in an external magnetic field [7].

Magnetic Fe_3_O_4_ nanoparticles (MNPs) are often functionalized with various coatings or are embedded in a polymer or organic matrix to improve their biocompatibility, colloidal stability, and increase their circulation time in biological media; MNPs are also modified with target agents or drugs specific for tumor cells [9,10,11]. For targeted delivery, MNPs can be modified with specific antibodies or their synthetic analogues—nucleic acid aptamers with high selectivity, small size, and low immunogenicity. Coating with aptamers improves MNPs’ biocompatibility, colloidal stability and increases the circulation time in biological media [12]. Furthermore, functionalized MNPs are used as contrast agents in modern diagnostic methods, such as magnetic resonance imaging (MRI), positron emission tomography (PET) and single photon emission computed tomography (SPECT) [10,13,14]. Among the many therapeutic uses of magnetic particles, their application in localized heating of tumors, i.e., hyperthermia, is the most widely known [15,16,17]. 

Recently, gene transfection methods are being further developed with the use of magnetic nanoparticles—magnetofection [7,18]. One of the most studied areas of medicine is tumor targeted drug delivery [12,13,19,20,21]. Moreover, targeted drug delivery is often used in conjunction with hyperthermia [17,22,23,24,25,26]. 

Optimization of the nanoparticles-based therapy and design of efficient drug carriers could be performed using a multiscale computational framework, developed by Li Y. and coauthors [8,21]. This approach is useful for understanding the detailed mechanisms behind the NP-mediated drug/gene delivery process, and the microvascular transport of NPs in blood flow, namely, their adhesion to vessel walls in flow, as well as cellular uptake of NPs [21].

However, the main interest in magnetic particles is most likely due to the possibility of combining methods for diagnosis and therapy [27,28]. Thus, this article will consider magnetic particles alone, and in complexes with aptamers, as diagnostic and therapeutic agents in cancer.

## 2. Aptamers

Nucleic acid and peptide aptamers have enormous potential as probes for molecular recognition. Single-stranded small DNA and RNA aptamers fold into well-defined three-dimensional structures, and show high affinity and specificity for their targets [29]. Functionally, aptamers are analogues of antibodies, but in many respects, aptamers are superior. They are selected through an in vitro evolution process in a few days, without prior knowledge of the cell-specific biomarkers to live cells, tissues, viruses, bacteria, proteins, and small molecules [29,30,31,32,33,34,35,36,37,38,39]. The resulting aptamers are chemically synthesized with simple chemical procedures in high purity and at low cost; therefore, they are considered to be synthetic chemical products, rather than biological. The production of aptamers is significantly cheaper than the production of monoclonal antibodies, and due to their high selectivity, small size (5–30 kDa), and low immunogenicity, aptamers could be used for therapeutic purposes as delivery agents [29,40]. 

## 3. Characterization of Magnetic Nanoparticles

**Figure 1:**
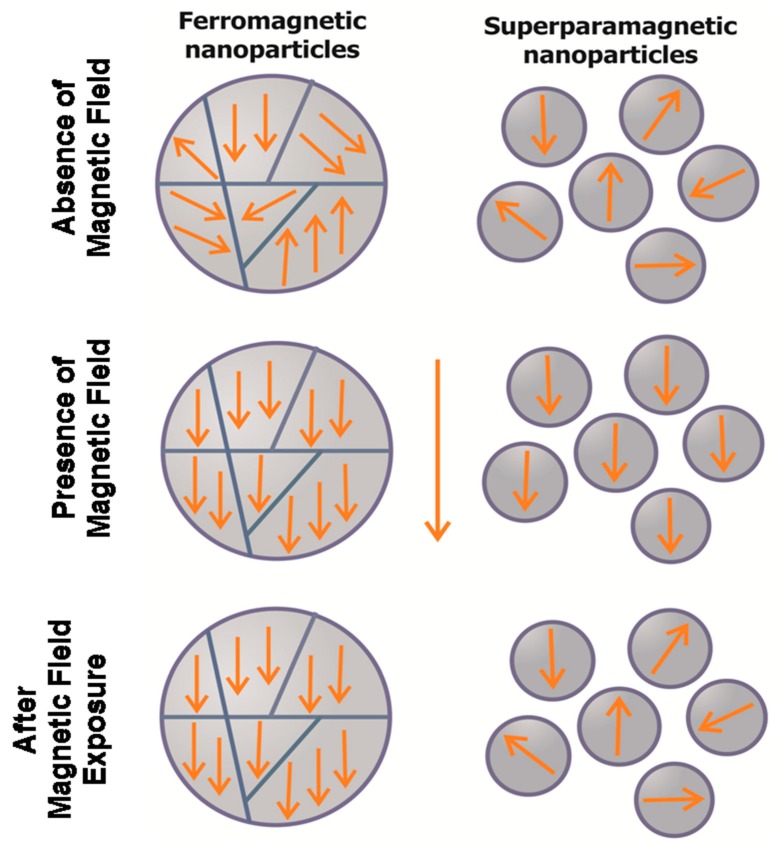
Illustration of superparamagnetic and ferromagnetic particles in the presence and absence of a magnetic field (MF), and after exposure to a MF. In the presence of an alternating magnetic field, the magnetic moment of both superparamagnetic and ferromagnetic nanoparticles are aligned. Upon removal of the magnetic field, the nanoparticles maintain the net magnetization.

Magnetic nanoparticles are nanostructures with highly active surfaces and size-dependent physical properties, such as magnetic characteristics. Typical magnetic properties include ferromagnetism and superparamagnetism, which occur in the transition from substance to nanostate (Figure 1). Superparamagnetic particles are magnetic in the presence of a magnetic field; without magnetic field exposure, their magnetic moment is zero. The magnetic properties of nanoparticles influence the chemical composition, type of crystal lattice, shape of the particles, and their interactions with neighboring particles.

**Figure 2:**
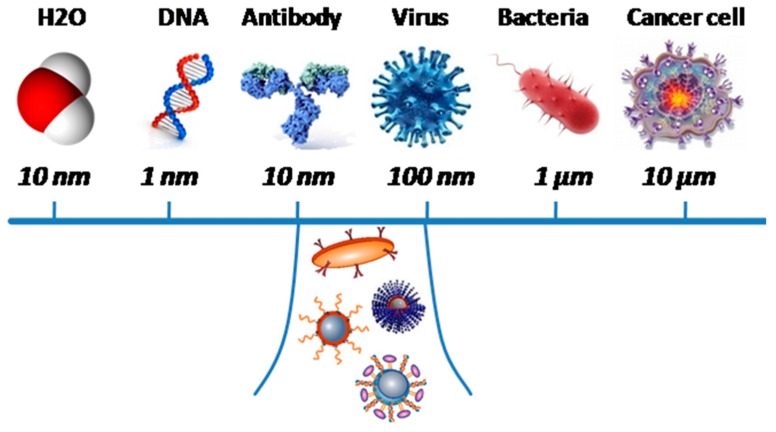
Size scale of MNS as compared to biomolecules.

Nanoparticle form can vary significantly. At present, most studies are focused on the properties and capacity of synthesis and use of anisotropic magnetic particles. Furthermore, due to their nano-size (less than 100 nm), which is comparable to cell size (10–100 micrometers), viruses (20–100 nm), proteins (5–50 nm) and DNA (width 1 nm, 10–100 nm), nanoparticles may be able to approach biological objects, in order to interact and communicate with them (Figure 2). An important aspect, apart from size, is the surface charge of MNPs, which must be neutral for greater specificity. This is because positively charged particles tend to non-specifically bind to cells; in contrast, neutral MNPs can maximize circulation time in the bloodstream, which is important for their use in biomedicine as therapeutic and targeted drug delivery agents [15,41,42,43,44]. Physical characteristics of magnetic nanoparticles significantly influence their effectiveness in vivo. Morphology, surface charge and particle size are considered important determinants of the pharmacokinetics, biodistribution and toxicity of the particles in the body [1].

By now there are a variety of magnetic nanoparticles actively used in biomedicine: iron-based oxides, metals such as Co, Fe, Ni, ferrites such as MgFe_2_O_4_, SoFe_2_O_4_, MnFe_2_O_4_, as well as multifunctional MNPs with two or more different functional units, such as Au–Fe_3_O_4_, FePt–CdS, and Fe_2_O_3_–carbon nanotube, which can be synthesized through seed mediated growth. In a heterogeneous nanostructure, such as Fe_2_O_3_-carbon MNP, each unit exhibits its unique magnetic, optical, or electronic properties [1,15,28,41,42,43,45,46,47,48].

It should be noted that the oxide particles have weaker magnetic properties than the metal-based nanoparticles; they are, however, more resistant to oxidation. Currently, nanoscale particles of iron oxide are most widely applied in biomedicine, which is due to their low toxicity and the high stability of their magnetic characteristics [49,50].

## 4. Production of Magnetic Nanoparticles

There are, therefore, several strategies for the synthesis of MNPs of particular size, functionality, and stability:

Physical methods include gas-phase deposition and electron beam lithography. However, these methods suffer from their inability to keep particle size to the nanometer scale [10]. Other studies [47,51] describe a lithography and magnetron sputtering technique, which can obtain a particle diameter of 1 to 2.5 μm, consisting of three layers: a layer of permalloy, which is located between two layers of gold.

Wet chemical preparation methods include sol−gel synthesis, oxidation methods, chemical coprecipitation, hydrothermal reactions, flow injection synthesis, electrochemical methods, aerosol/vapor phase methods, sonochemical decomposition reactions, supercritical fluid methods, and synthesis using nanoreactors [52,53,54].

Chemical routes are preferred, because they can synthesize MNPs with uniform composition and size [52,53,54,55]. The most common synthetic strategy involves aqueous precipitation of iron salts with in situ or post-synthesis addition of surfactant. The thermal decomposition/reduction method has gained considerable attention, because this technique offers fine control over the final particle size, shape, and crystal structure in comparison to other methods, and is also scalable [55].

Biological and microbiological methods are generally simple, versatile, and efficient, with appreciable control over composition and particle geometry of the resulting material [56,57]. The used microorganisms are bacteria, actinomycetes, fungi and algae. The synthesis location of nanoparticles can be intracellular or extracellular. For example, magnetic nanoparticles can be isolated from magnetotactic bacteria cells [58].

## 5. Coatings and Functionalization of MNPs

For diagnostic and therapeutic applications of magnetic nanoparticles, their surfaces are usually modified with various coatings and functional biological molecules to acquire stability in solution, to increase the biocompatibility, and decrease the toxicity of magnetic materials to the living organism.

**Figure 3:**
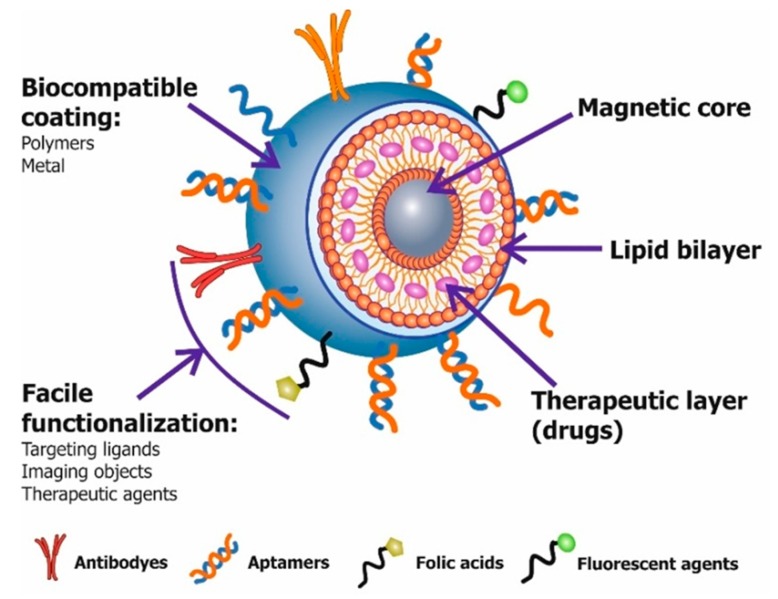
Schematic illustration of a multifunctional magnetic nanoparticle structure with different types of coatings, target ligands and imaging agents. Therapeutic drugs can be embedded in the coating, or conjugated on the surface.

Coatings of magnetic nanoparticles use many organic and inorganic substances, such as dextran, polyethylene glycol (PEG), starch, chitosan, liposomes, gold, polyvinylethanol, biotin, heparin, etc. (Figure 3) [52,59]. Polyethylene glycol (PEG) is one of the most common polymeric ligand MNP surface coatings [17,60]. PEG coating improves the dispersion of MNPs in biological environments, and increases circulation time in blood, because they are not easily recognized by the reticuloendothelial system (RES) [61]. PEG reduces nanoparticles absorption by macrophages, and due to polar and nonpolar groups, promotes effective penetration through the cell membrane [48]. Another widely used coating is the dextran polymer; this is because of its biocompatibility and polar interactions [52,59]. Silica coating on MNPs is popular due to ease of synthesis and its stability in aqueous conditions [52,62,63]. MNPs modification by phospholipid layer (liposome formation) is also a typical method. The liposomes structure is similar to biological membranes and determines their biocompatibility and efficacy in the targeted delivery systems [60,64]. Recently, more attention has been given to the preparation of magnetic nanoparticles with carbon coatings, due to their advantages over polymers or silica, namely, a much higher chemical and thermal stability [65]. Inorganic metals, such as gold, protect the MNP’s magnetic core against oxidation, corrosion, aggregation and impart its biocompatibility. Due to their optical properties, they are used in localized surface plasmon resonance and surface-enhanced Raman scattering, and therefore, can also be used in magnetic resonance imaging as contrast or therapeutic agents [66].

After the nanoparticle is covered with a suitable coating, its surface is further modified with various functional groups, such as azido, amino, carboxyl, sulfhydryl, hydroxyl, imide, thiol, and others that allow the nanoparticles to bind to target biomolecules or therapeutic agents [42,54,65,67]. As such, peptides, oligonucleotides, antibodies, polysaccharides, and small molecules such as acids, are commonly used ligands (Figure 3) [7,17,42].

Cancer studies have found that antibodies and aptamers are highly specific biomolecules that help the nanoparticles concentrate locally on the tumor target, by binding to specific receptors on the cell surface. Aptamers as targeting molecules have advantages over antibodies, which include their small-size (15 kDa), low immunogenicity and ease of synthesis without batch-to-batch variations and ease of chemical modifications [43,68]. Aptamer coated nanoparticles have been used for photodynamic [69] and photothermal [70,71] cancer therapy, which selectively destroys generations of cells by reactive oxygen species, through the use of light and a photosensitizer. Aptamers escort nanoparticles to cancer cells, improve their accumulation in tumor, and selectively induce photo- or thermal damage of abnormal cells [72,73]. 

The use of acids for targeted delivery of nanoparticles has also been well documented. For example, folic acid is a water-soluble vitamin B6, which participates in rapid cell growth and division processes, especially during embryonic development [74].

Cancer studies also found an over expression of folate receptors on the surface of tumor cells; therefore, nanoparticles functionalized with folic acid bind with high affinity to tumor cells [43]. Carbohydrates also established themselves as target ligands; for example, asialoglycoprotein receptor (ASGP-R), present mostly in hepatocytes [75,76] readily binds galactose, mannose and arabinose; thus, these carbohydrates can be used as agents for targeted delivery to the liver [43,77].

## 6. Application in Diagnostics

Over the last few decades, magnetic nanoparticles have been widely used in the diagnosis of various diseases, with a focus on their use in the treatment of cancer. Due to their small size, magnetic properties and multifunctionality, nanoparticles are used in diagnostic methods such as scintigraphy, single photon emission computed tomography (SPECT), positron emission tomography (PET), magnetic resonance imaging (MRI), and Raman spectroscopy [14,78,79,80,81]. The most popular methods in the diagnosis of cancer are SPECT, PET and MRI. SPECT and PET have high-sensitivity in tracking biological events, but they have poor spatial resolution [81]. MRI has excellent soft tissue contrast and multi-dimensional functional, structural and morphological information, but it suffers from low sensitivity [81]. Therefore, modern diagnostic methods are continually under development, increasing their accuracy by means of various agents. Currently, MRI is one of the prospective and most developed methods for utilizing magnetic nanoparticles in highly sensitive diagnostics.

## 7. Magnetic Resonance Imaging

To date, MRI is used almost universally. This method is a highly accurate, sensitive and non-invasive diagnostic method that allows for the diagnosis of various diseases in their early stages. The main advantage of MRI is high spatial resolution and contrast in soft tissue in comparison to other imaging techniques [82]. MRI magnetic nanoparticles are used as contrast agents because they are capable of increasing the relaxation time of hydrogen protons, thereby increasing signal repeatability [41,78,79,80,81]. The literature describes examples of the use of nanoparticles of various compositions as contrast agents [27,79,83]. Total MRI can be used for two different classes of magnetic nanoparticles: ferromagnetic iron oxide particles, and ultra-small superparamagnetic iron oxide particles [55,84]. Typically used in MRI are complex functionalized MNPs, consisting of a magnetic core, amplified signals of the magnetic field-stabilizing coatings, and surface biological agents, for specific accumulation of nanoparticles [17,80]. Additional agents, such as fluorescent labels and radionuclides with magnetic nanoparticles, can also be used to obtain a more accurate diagnosis [82]. Many articles describe MRI application in combination with magnetic nanoparticles. Sun et al., reported use of Fe_3_O_4_ MNPs coated with peptide, and demonstrated by MRI, the in vivo ability of tumor-specific targeting [63]. MRI showed that the accumulation of (RGDyK)–MC–Fe_3_O_4_ NPs in the tumor was mainly localized on the integrin-expressing vasculature and on tumor cells with little or no macrophage uptake [85]. Kievit F.M. et al., described using iron oxide nanoparticles coated with co-polymer of chitosan and polyethylene glycol, and modified with a fluorescent dye functionalized with an antibody, against a Neu breast cancer receptor [86]. The results showed that targeted nanoparticles reduced MRI signal intensity up to three times, in comparison to the same modified nanoparticles with nonspecific antibody. 

In another study, aptamer-modified thermo-sensitive liposome TSLs–AS1411 was used as an efficient magnetic resonance imaging probe. Gd–DTPA was encapsulated into an optimized thermo-sensitive liposome (TSL) formulation, and then conjugated with AS1411 for specific targeting against tumor cells that overexpress nucleolin receptors. MRI revealed the absence of liposome toxicity and the presence of high biocompatibility. Moreover, the AS1411 targeted TSLs showed an enhanced imaging effect on targeted cells in response to a mild hyperthermic treatment [87]. Magnetoliposomes are also used as multimodal contrast agents for molecular imaging [67].

## 8. Application in Therapy

### 8.1. Chemotherapy or Drug Delivery

Drug delivery agents are widely used in therapy due to their unique properties and ability to remote control functionalized magnetic nanoparticles. One of the main advantages of targeted drug delivery is the increase of the local concentration in the target, which significantly increases the effectiveness of therapy.

**Figure 4:**
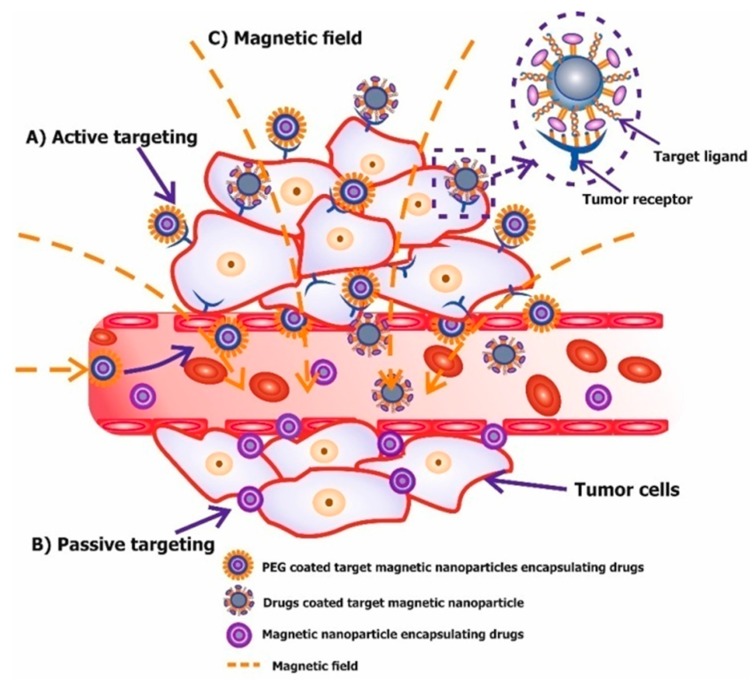
Modes of tumor-targeting magnetic nanoparticles. (**A**) Passive targeting (enhanced permeability and retention (EPR) effect) of magnetic nanoparticles. Nanoparticles reach tumor cells selectively through the leaky vasculature surrounding the tumors; (**B**) Active (molecular targeting). Ligands (aptamers, antibodies, peptides, small molecules, etc.) linked with magnetic nanoparticles that bind to receptors overexpressed by tumor cells; (**C**) Magnetic targeting.

Targeted delivery of nanoparticles takes place by passive and active targeting and by remotely controlling the alternating magnetic field (Figure 4) [43,52]. 

Passive delivery is due to the enhanced permeability and retention (EPR) effect of the tumor, which is important for selective accumulation of the nanoparticles. However, the use of passive targeting is limited because of the individual characteristics of vessels in different types of tumors [43,88]. The solution to this problem includes application of active targeting by modifying the surface of magnetic nanoparticles with drug substances and specific moieties, which selectively bind only to receptors found on tumor target cells. This strategy simultaneously provides highly efficient delivery. Moieties such as antibodies, aptamers, peptides and small molecules are used to target tumor biomarkers [10,68]. Active targeting may also be achieved, due to the ability of magnetic nanoparticles to respond to an external magnetic field. The magnetic force produced by the gradient actively attracts particles into the tumor space (through the comprised vasculature) and helps in subsequent retention [89]. It should be noted that the various methods of delivery of therapeutic magnetic nanoparticles can be used simultaneously.

Jalalian et al. used antibody-functionalized MNPs [90], previously described by Huang C. et al. [91]; in one of the studies, epirubicin loaded 5TR1 aptamer functionalized superparamagnetic iron oxide nanoparticles (SPION) was administered to mice with colon cancer. This process led to a significant reduction in tumor growth [90]. Hadjipanayis C.G. et al. used bioconjugates of anti-epidermal growth factor receptor (EGFR) deletion mutant antibody with iron oxide nanoparticles (EGFRvIIIAb–IONPs) for targeted imaging and therapeutic treatment of glioblastoma [92]. EGFRvIII specific binding was achieved by creating polyclonal rabbit antibodies EGFRvIIIAb. According to the study, there was a significant number of survivors undergoing therapy with EGFRvIIIAb-IONPs, while using only IONPs or EGFRvIIIAb did not increase survival [92].

**Figure 5:**
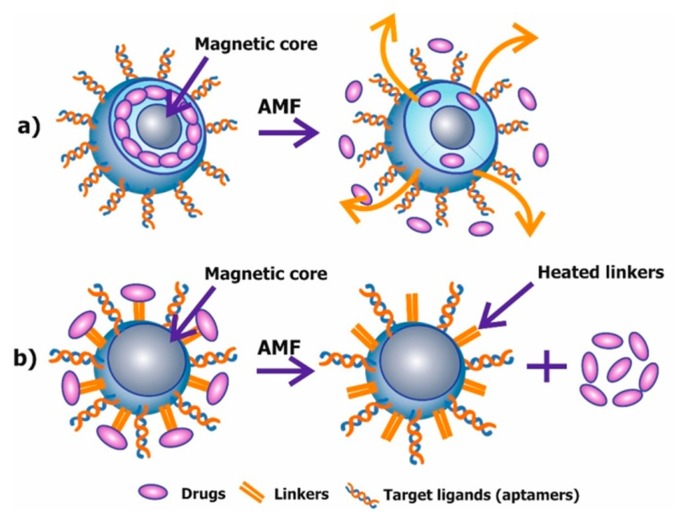
Schematic representation of the two mechanisms of controlled drug delivery using a magnetic field based hyperthermia. (**a**) Magnetic hyperthermia-based controlled drug delivery through enhanced permeability; (**b**) Magnetic hyperthermia-based controlled drug delivery through bond breaking (linkers).

Fazilati M. synthesized doxorubicin (DOX) loaded folate-coated magnetic Fe_3_O_4_ nanoparticles (MNPs) [93]. Folic acid was used because folate binding receptors are overexpressed in most human tumors, especially in ovarian cancer cells. Their research showed that folic acid modified MNPs amplify DOX-induced apoptosis in human ovarian cancer cell lines, with a sharp decrease in levels of Bcl–2 and survival rate of cancer cells, and increase in expression of caspase–3. Folate modified magnetic nanoparticles were also investigated in the works of Gunduz U. et al. [94] and Chen H. et al. [95]. Iron-tagged single-walled carbon nanotubes (SWCNTs) conjugated with Endoglin/CD105 antibody, with or without DOX, were used by Faraj A.A. [96]. Their therapeutic effects were tested in mouse breast cancer cells. Investigation of DOX-loaded SWCNTs conjugated with antibodies showed DNA damage, oxidative stress and a significant increase in apoptotic tumor cells [96]. Active targeting using a magnetic field and allocated hyperthermia-based controlled drug delivery, based on conjugating a drug molecule to the MNP via a linker and applying AMF, lead to the release of the drug molecule, due to the heating of a linker molecule attached to the surface of the NP (Figure 5a) [97]. Derfus A.M et al., describes an example where a nucleic acid duplex was used as a heat-labile linker [97].

It is also possible to release the drug within the polymer matrix of the nanoparticles, encapsulated with MNPs upon application of alternating magnetic field (AMF)/electromagnetic field (EMF) (Figure 5b) [98].

In a study by Alexiou C. et al. iron oxide nanoparticles covered with starch derivatives with phosphate groups were used as carriers for drugs such as cytostatic mitoxantrone [99]. Due to the magnetic field gradient, the nanoparticles accumulated in the tumors and provided the cytostatic effect [99]. Similarly, the delivery of doxorubicin hydrochloride was carried out by Huang C. et al. [91]. Magnetoliposomes have been extensively studied, with avid interest, for various biomedical applications. For controlled drug release from liposomes, researchers use localized heating and/or mechanical guidance MNPs. For example, Qiu D. and An X.Q. presented a drug-delivery system based on liposomes, which enclose hydrophobic MNPs in their lipid bi-layer, with calcein as a model hydrophilic drug [100]. For this study, liposomes were controlled with an alternating magnetic field [100].

In his work Y.J. Chen et al., used polyethylene glycol-stabilized bi-layer-decorated magnetoliposomes (dMLs) loaded with doxorubicin hydrochloride [101]. The results showed that dMLs in conjunction with radiofrequency (RF) electromagnetic field caused 90% cell death in Huh–7 hepatocellular carcinoma [101]. Other researchers have developed liposomes consisting of phospholipids, iron oxide magnetic nanoparticles and thermo-sensitive block copolymers [102]. These hybrid liposomes release the drug when exposed to an AMF more actively than in the absence of AMF. The magnetically enhanced drug release is attributed to the transition of the thermos-sensitive segment of copolymers [102,103].

Magnetofection is another method for delivery of therapeutic agents to transfect magnetic nanoparticles associated with DNA vectors into cells by an applied external magnetic field. Genetic material delivered to the target cell can serve plasmid DNA, double-stranded DNA, mRNA, and siRNA oligonucleotides [52]. One of the most promising candidates for gene therapy is siRNA (small interfering RNA). They are associated with magnetic nanoparticles by a disulfide bond, and can be easily released by using enzymes. Small interfering RNA is capable of suppressing expression of certain genes by inhibiting protein translation in the cytoplasm [28]. Magnetofection technology, in which a magnetic field creates oscillations of the magnetic particles, contributes to more efficient absorption of cells [104]. Prosen L. et al., investigated antitumor effectiveness of SPIONs–PAA–PEI–pDNA (magnetofection complexes) with plasmid DNA encoding short hairpin RNA (shRNA) against Mcam (pDNAanti-MCAM) [105]. Treatment with magnetofection complexes, with alternating magnetic field after three applications, led to a significant reduction in tumor volume. Detailed magnetofection is described in the works by Plank C. et al. and others [105,106,107]. Modified particles with tetrandrine were used to trigger apoptosis of lung cancer cells A549. The apoptotic effect, according to the authors, was due to inhibition of the expression of anti-apoptotic proteins bcl–2 and bcl–xL [108].

In recent years, attention has been brought to bacterial magnetic nanoparticles (BMPs). For example, Guo L. et al., produced functionalized bacterial magnetic nanoparticles with magnetic drug targeting and tumor bio-targeting properties, through a combination of doxorubicin and galactose target ligand, specific for hepatocellular carcinoma, to the membrane of bacteria [109].

### 8.2. Magnetic Mediated Hyperthermia

**Figure 6:**
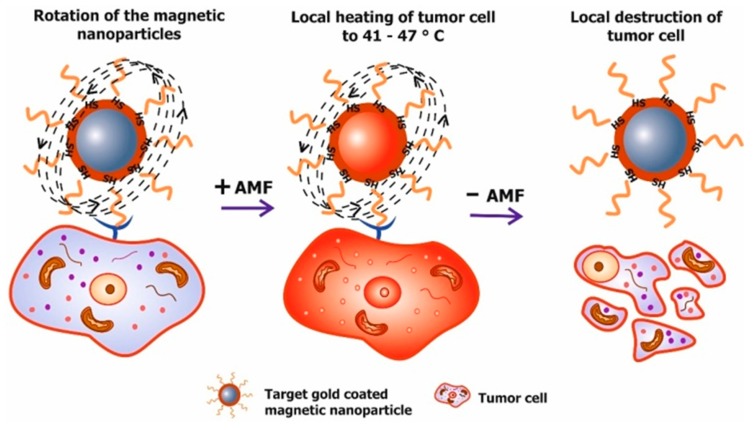
Principle of magnetic mediated hyperthermia. Targeted magnetic nanoparticles delivered to tumor cells are exposed to an alternating magnetic field (AMF). Afterword, AMF energy is converted into heat by the magnetic nanoparticles, which leads to local heating of tumor cells between 41 and 47 °C.

Tumor cells are more sensitive to heat than normal tissue, as a result, a method was developed for selective thermal destruction of tumor cells, by means of the transformation of alternating magnetic field (AMF) energy into thermal energy by functionalized magnetic nanoparticles, which is defined as heating the tissue in the range of 41–47 °C (Figure 6) [59,110].

Energy conversion nanoparticles are effective because the rate of adsorption is correlated with the heating rate of particles placed in the alternating magnetic field. In the work by Liu X.L. et al. [111], investigations into the dependence of the adsorption rate on the size of the nanoparticles showed, that the optimum rate of adsorption for hyperthermia was observed in particles that were 18 nm. Hyperthermia, or rather magnetic mediated hyperthermia, is advantageous because heating is localized to the center of the tumor tissue, due to the selective binding of functionalized magnetic nanoparticles with tumor cells, and the use of AMF. Many studies have described magnetic mediated hyperthermia as an effective method of treatment [16,103].

In addition, different variants of local magnetic hyperthermia are currently being actively developed. One variant is based on a magnetic material which is injected into the affected area, and heated from the outside by means of electromagnetic radiation, ranging from 100 to 800 kHz, with little absorption by other tissues, but strongly interacts with ferromagnets and superparamagnets [46].

Kossatz S. et al. reported the use of modified superparamagnetic iron oxide nanoparticles, such as (MF66), Nucant 6L (MF66-N6L), doxorubicin (MF66-DOX) or both (MF66-N6LDOX), together with magnetic hyperthermia, for treatment of breast cancer. The results showed that MF66-DOX, and MF66-N6LDOX combined with hyperthermia, were more toxic to breast cancer cells than each corresponding NP with ligands. There was significant inhibition of tumor growth and, in many cases, complete disruption [22].

A similar approach was reviewed in an article by C.A. Quinto et al., where SPIOs was synthesized with a phospholipid–polyethylene glycol (PEG) coating, and loaded with doxorubicin for joint hyperthermia and chemotherapy of tumor diseases [112].

F. Mohammad and N.A. Yusof obtained and investigated the probe doxorubicin (Dox) loaded gold-coated superparamagnetic iron oxide nanoparticles (SPIONs@Au) for combination therapy of cancer, by means of both hyperthermia and drug delivery [23]. The probe generates local hyperthermia in accordance with the external magnetic field, while the outer controlled delivery of conjugated drug can be achieved from the oscillation of particles with the help of same field; this system effectively induced tumor cell death [23].

J. Kolosnjaj-Tabi et al., created PEG-coated iron oxide nanocubes to mediate mild tumor magnetic hyperthermia treatment [113]. Heat-generating PEG-coated iron oxide nanocubes showed interference with the tumor’s extracellular matrix, and the potential to destroy the matrix under magnetic influence, which leads to a decrease in tumor growth [113].

Other authors have reported the use of a multifunctional platform for drug delivery and magnetic hyperthermia of malignant tumors, where the foundations were carbon-encapsulated magnetic colloidal nanoparticles with silica coating (MCN@C/mSiO_2_), and the rattle-type structured magnetic mesoporous silica nanoparticles (MCN/mSiO_2_). The MCN@C/mSiO_2_ nanoparticles exhibited higher magnetic hyperthermia ability compared to the MCN/mSiO_2_ nanoparticles, but the MCN/mSiO_2_ nanoparticles had higher drug loading capacity. Research has shown that drug release from the two types of complexes were temperature-dependent [26].

### 8.3. Mechanical Destruction of Cells and Triggering of Apoptosis 

Studying the mechanisms of cell function, and most importantly, the processes of signal transduction, are difficult tasks for researchers. Magnetic nanoparticles can be successfully applied to magnetically launched apoptosis and mechanical destruction of cell membranes [47,51,108,114,115,116] (Figure 5). These effects are important in cancer treatment, because the sensitivity of cancer cells to apoptosis in vivo is significantly reduced.

The work by Kim D.-H. (2010) explores the interaction of magnetic microdiscs with cancer cells (glioma multiform) in vitro [114]. Permalloy discs with an outer gold layer were covered with antibody molecules specific for IL132R; this is because IL132R is overexpressed on the surface of glioma cells, and as a result, serves as a target. When an external alternating magnetic field is applied, such microdiscs begin to vibrate, transmitting mechanical vibrations in the cell. The researchers concluded that mechanical stimulation of cells by microdiscs is accompanied by two important effects: the cell membrane violation of integrity, and launch of the self-destruction program of the cell. The study showed that a ten-minute exposure time to a magnetic field frequency of several tens of Hz was enough to destroy 90% of cancer cells in vitro [114].

The possibility of using magnetic nanoparticles to induce apoptosis was further confirmed by the work of Cho M.H., [117] where apoptosis was performed in vitro on colon cancer cells and in vivo in zebra fish. Antibodies to a cell death receptor were immobilized on the surface of particles consisting of iron-doped and zinc [117]. 

Apoptosis has also been triggered by using magnetic nanoparticles modified with folic acid. Initiation of apoptosis and a decrease in proliferation of BEL–7402 liver cancer cells in a magnetic field, with a frequency of 100 Hz, was demonstrated [115].

**Figure 7:**
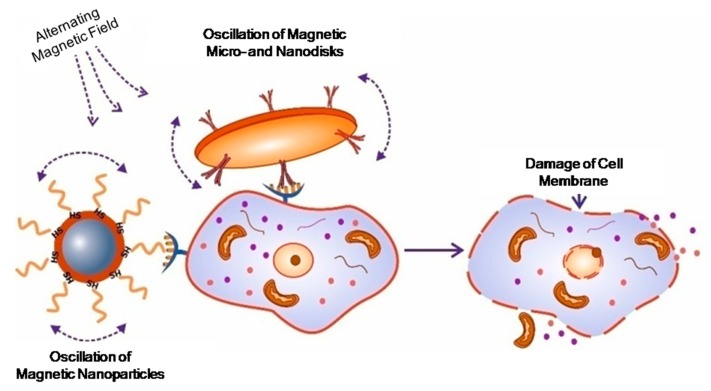
The concept of targeted magnetomechanical cancer-cell destruction using magnetic nanoparticles with different shape.

In the works by Kim P.D. and Zamay T.N. [116,118], the antitumor effect of DNA aptamer modified nickel magnetic microdisks coated with gold, was investigated in vivo and in vitro. It was found that the DNA aptamer modified microdisks, under the influence of a rotating magnetic field, are capable of causing death of ascites cells, in cell culture or in an organism [116,118] (Figure 7). 

The other study [119] demonstrates that aptamer-functionalized 50 nm gold-coated superparamagnetic nanoparticles can be used for targeted magnetodynamic therapy in vitro and in vivo (Figure 7).

Thus, the triggering of apoptosis using magnetic particles bound to antibodies or aptamers and drugs, is a relatively new, but very positive, direction of further research.

## 9. Biodistribution and Toxicity of Gold and Magnetic Nanoparticles

Important factors in considering the use of nanoparticles in medicine are their behavior in the body and their potential toxicity; these are particularly significant when considering long-term therapy. The components that make up the nanoparticles and their effects are equally important; therefore, defined parameters must be set for possible effects such as: hematotoxicity, activation of the complement system, carcinogenicity, teratogenicity, immunogenicity, etc. Furthermore, it is also crucial to examine the conjugates of the nanoparticles [120].

Despite the biocompatibility of magnetic nanoparticles and their non-toxicity, their elimination is problematic because of their potential to accumulate in the body. It is recommended that particle sizes should be small enough to avoid capture by the reticuloendothelial system and phagocytes forming part of the immune system; however, the nanoparticles should have a sufficiently large enough size to avoid renal clearance.

The literature reports that larger nanoparticles are collected from blood more rapidly than smaller nanoparticles, and it was found that, ideally, particle sizes should range between 5.5 nm and 200 nm [63]. However, it was also shown, that for particles smaller than 40 nm in diameter, biodistribution and half-life are determined by the coating material, rather than the average hydrated size. Many studies reported that more than 75% of the magnetic nanoparticles were absorbed by the reticuloendothelial system (RES), mostly by the liver, and it has been suggested that the Kupffer cells can convert most of the iron in ferritin [87]. In general, particle size of 20 to 30 nm is considered optimal. Nanoparticles in this size range are readily absorbed by endocytic vesicles, whereas particles larger than 150 nm cannot enter cells by endocytosis; instead, they are phagocytosed by macrophages and later transported in the RES. Capture processes are enhanced because of insufficient known blood proteins. In addition to size, surface charge of nanoparticles plays a decisive role for the half-life period in blood. Positively charged particles usually adhere non-specifically to cells. However, strong negatively charged particles are also harmful, because this ultimately increases the degree of particle uptake by the liver. Thus, it is considered that magnetic nanoparticles with a neutral surface charge have the highest circulation time in the bloodstream [44].

## 10. Conclusions

The high toxicity and low efficiency of anticancer drugs demands the need for the development of new drugs and treatment technologies. In recent years, non-standard anticancer therapy tools have been developed based on nanotechnology, involving physical methods of tumor destruction using nanoparticles, which have unique properties. The disadvantage of such methods in combination with the nanoparticles, is the insufficiently high specificity of physical action factors, whereby nanoparticles accumulate in all tissues, not only in the tumor, and the use of physical methods exposure (magnetic field and laser irradiation) causes damage to the tissue surrounding the tumor.

It has become apparent that increased efficiency of nanoparticle-based cancer magnetotherapy can be achieved by using tumor recognizing biomolecules which escort MNPs to their targets. Currently, biomolecules such as monoclonal antibodies are often used. Along with antibodies, aptamers are becoming promising agents for targeted delivery of nanoparticles. Aptamers as well as monoclonal antibodies bind with high specificity to desirable biological targets. To date, several effective aptamer-based magnetic nanoplatforms have been developed for a targeted cancer therapy; the most important works are summarized in Table 1.

Thus, magnetic nanotheranostics, on the basis of various ligand and/or drug functionalized nanoconstructions, opens a new venue for complex differential diagnostics, and metastatic cancer therapy.

## Figures and Tables

**Table 1:** Magnetic nanoparticles and their applications for a targeted cancer theranostics.

Materials	Size	Coatings	Antitumor Drugs	Linkers	Targeted Ligands	Applications	Reference
Gd-DTPA	123.2 nm	Thermo-sensitive liposome (DPPC)	-	Carboxyl groups	Aptamers	MRI	[87]
Iron oxide (magnetite)	51.43 ± 4.52 nm	-	Epirubicin	Amine, carboxyl groups	Aptamers	Targeted chemotherapy MRI	[90]
Iron oxide (magnetite)	10 nm	-	Dextran	Thiol groups	Aptamers	Magnetic hyperthermia	[24]
Iron oxide (magnetite)	12 ± 3 nm	-	Doxorubicin	Thiol groups	Aptamers	Magnetic hyperthermia computed tomography	[22]
Ironoxide (magnetite)	15.4 nm	Gold Polyethylene-glycol	-	Amino and thiol groups	Aptamers	Targeted magnetic hyperthermia	[25]
Nickel magnetic microdisks	500 nm	Gold	-	Thiol groups	Aptamers	Mechanical destruction of cells and triggering of apoptosis	[116,118]
Iron oxide (magnetite)	50nm	Gold	-	Thiol groups	Aptamers	Apoptosis induction via fibronectin binding aptamers	[119]
Iron-tagged single-walled carbon nanotubes	200–300 nm	Polyvinyl-pyrrolidone	Doxorubicin	Carboxyl groups	Antibodies	Targeted chemotherapy MRI	[96]
Ironoxide (magnetite)	10 nm	Polyethylene-glycol	-	Carboxylate and Amino groups	Antibodies	Targeted therapy MRI	[92]
Ironoxide (magnetite)	40 nm	Chitosan and polyethylene-glycol	-	Amino and thiol groups	Antibodies	MRI	[86]
Mn-Zn ferrite MNCs	42.3nm	Polyethylene-glycol	-	Carboxyl groups	Сyclic tripeptide of arginine-glycine-aspartic acid	Targeted magnetic hyperthermia MRI	[17]
Ironoxide (magnetite)	5 nm	Lipid bilayer (DPPC/PEG750-PE)	Doxorubicin	-	-	Targeted chemotherapy controlled by electromagnetic fields	[101]
Ironoxide (magnetite)	6.8 nm	Gold	Doxorubicin	Cystmolecules	-	Chemotherapy magnetic hyperthermia combinatorial treatment	[23]
Iron oxide (magnetite, maghemite)	16.1 nm	Mesoporous silica	Doxorubicin	-	-	Targeted chemotherapy and magnetic hyperthermia	[26]
Iron Oxide Nanocubes	19 nm	Polyethylene-glycol	-	-	-	Magnetic hyperthermia MRI	[113]
Ironoxide (magnetite)	14 nm	Phospholipid-Polyethylene-glycol coating	Doxorubicin	-	-	Chemotherapy-magnetic hyperthermia combinatorial treatment	[112]
Ironoxide (magnetite)	5 nm	-	Cytostatic mitox-antrone	Phosphate groups	-	Targeted chemotherapy controlled by strong inhomogeneous magnetic field	[99]
